# The role of ASXL1, SRSF2, and EZH2 mutations in chromatin dysregulation of myelodysplastic neoplasia and acute myeloid leukemia

**DOI:** 10.1038/s41375-025-02657-9

**Published:** 2025-06-25

**Authors:** Hosang Yu, Junshik Hong, Dong-Yeop Shin, Chul-Hwan Lee

**Affiliations:** 1 Department of Pharmacology, Seoul National University College of Medicine, Seoul, Republic of Korea; 2 Department of Biomedical Sciences, Seoul National University College of Medicine, Seoul, Republic of Korea; 3 Department of Internal Medicine, Seoul National University College of Medicine, Seoul, Republic of Korea; 4 Cancer Research Institute, Seoul National University, Seoul, Republic of Korea; 5 Center for Medical Innovation, Biomedical Research Institute, Seoul National University Hospital, Seoul, Republic of Korea; 6 Seoul National University Hospital, Seoul, Republic of Korea; 7 Ischemic/hypoxic Disease Institute, Neuroscience Research Institute, Seoul National University College of Medicine, Seoul, Republic of Korea; 8 Wide River Institute of Immunology, Seoul National University, Hongcheon, Republic of Korea; 9 The Institute of Molecular Biology & Genetics, Seoul National University, Seoul, Republic of Korea

**Keywords:** Myelodysplastic syndrome, Cancer epigenetics, Acute myeloid leukaemia

## Abstract

Mutations in chromatin-regulating genes play a critical role in the pathogenesis of myelodysplastic neoplasia (MDS) and acute myeloid leukemia (AML), as genetic mutations affecting chromatin structure and function are key drivers of these hematologic malignancies. Central to the discussion are key emerging genes such as *ASXL1*, *SRSF2*, and *EZH2*, which are recognized as adverse prognostic markers. Mutations in these genes, coupled with subsequent alterations in epigenetic mechanisms, disrupt normal gene expression by impairing histone modification and RNA splicing processes. Specifically, mutations in ASXL1 enhance removal of ubiquitylation at histone H2AK119, leading to altered gene expression and impaired hematopoietic stem cell differentiation. Mutations in SRSF2, an RNA splicing factor, alter RNA-binding specificity, inducing aberrant splicing of key genes such as *EZH2*. Loss-of-function mutations in EZH2 disrupt PRC2-mediated transcriptional repression, promoting leukemic progression. However, while the effects of these mutations are understood, treatment options for high-risk patients remain limited. Emerging strategies, such as venetoclax combined with hypomethylating agents, showing promise in mitigating the poor prognosis associated with these mutations. This review consolidates recent findings on these epigenetic regulators and their interactions, providing insights into the multifaceted mechanisms of leukemogenesis in the interest of inspiring targeted therapeutic strategies and bridging extant treatment gaps for MDS/AML.

## Introduction

Leukemia is a heterogeneous group of hematological malignancies originating from the transformation of hematopoietic stem cells (HSCs) into abnormal leukocytes. These malignancies can be broadly classified into lymphoid and myeloid neoplasms based on the lineage of the affected hematopoietic cells. Understanding the cellular phenotypes and broader characteristics of these malignancies is crucial for developing targeted therapies and improving patient outcomes.

In normal hematopoiesis, HSCs give rise to myeloid stem cells and lymphoid stem cells. Myeloid stem cells further differentiate into monocytes, megakaryocytes, neutrophils, and erythrocytes, cell types crucial for immune response, clot formation, and oxygen transport [1, 2]. Lymphoid stem cells differentiate into NK cells, T cells, and B cells, which are pivotal in adaptive immunity [2–4]. In leukemia, this differentiation process is disrupted, leading to the uncontrolled proliferation of abnormal cells and consequent impairment of normal hematopoiesis, resulting in symptoms such as anemia, infection susceptibility, and bleeding tendencies. Myelodysplastic neoplasia (MDS) and acute myeloid leukemia (AML) represent a continuum in the spectrum of leukemias, characterized by a complex array of gene mutations. Advancements in diagnostic technologies have broadened our understanding of these diseases, particularly revealing the importance of epigenetic factors and splicing factors in their development.

**Fig. 1:**
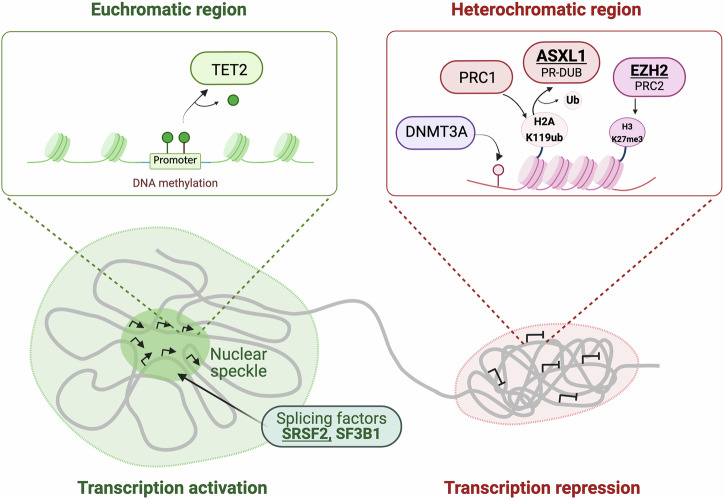
Chromatin modifiers that regulate gene expression are recurrently mutated in leukemia. Chromatin is largely classified into euchromatin and heterochromatin. Left (Euchromatic region): TET2-mediated DNA demethylation at promoter regions facilitates gene activation. Active transcription occurs within nuclear speckles, where splicing factors such as SRSF2 and SF3B1 are enriched, promoting efficient mRNA processing and gene activation. Right (Heterochromatic region): Polycomb repressive complexes (PRC1 and PRC2) contribute to chromatin compaction and transcriptional silencing by catalyzing histone modifications, including H2AK119ub and H3K27me3, respectively. ASXL1, a component of the PR-DUB complex, removes H2AK119ub, counteracting PRC1-mediated repression. Additionally, DNMT3A facilitates DNA methylation, further reinforcing transcriptional repression in these regions.

Mutations in *TET2*, *DNMT3A*, and *ASXL1* are considered central to the pathogenesis of MDS/AML [5]. These genes are frequently mutated in clonal hematopoiesis, with each being affected in more 18% of cases (Table 1). Such mutations represent early stages in leukemia progression and therefore provide ancillary support for its diagnosis. However, while use of these genes is prevalent in diagnostics, the pathogenesis of leukemia encompasses a wide array of genetic changes. The European LeukemiaNet (ELN) expert panel recognizes mutations in *ASXL1*, *EZH2* (histone modifiers), *SRSF2*, and *SF3B1* (splicing factors) as adverse MDS/AML risk factors [5]. This implies that the aggregation of mutations in this epigenetic and transcriptional machinery likely instigate abnormal gene expression contributing to the development of the disease [6, 7] (Fig. 1). Mutations in genes associated with epigenetic regulation are frequently identified in the early stages of leukemia, particularly MDS. The most commonly affected genes are *TET2* (23%), *DNMT3A* (18.4%), and *ASXL1* (18.2%). In addition to epigenetic regulators, splicing factor genes such as *SRSF2* (13.1%) and *SF3B1* (14.9%) also exhibit high mutation frequencies, highlighting their significant role alongside epigenetic modifiers (Table 1, top). These mutations can lead to additional epigenetic changes, contributing to the formation of a tumor-promoting environment. In addition, AML develops from MDS through the accumulation of further genetic mutations. Notably, in AML patients, genes such as *FLT3*, *NPM1*, and *NRAS* harbor mutations at higher frequencies compared to epigenetic factors (Table 1, bottom). Hence, the combination of facilitating epigenetic changes and subsequent mutations may drive the more aggressive progression observed in AML.

**Table 1:** Recurrent mutations in MDS/AML.

	Gene	Frequency	Function	Hotspot mutations	Deficiency
MDS	***TET2***	23%	DNA demethylase	Truncation mutations	Loss of function
	***DNMT3A***	18.40%	DNA methyltransferase	R882H/C/P/S/L/G(543/7538)	Aberrant DNA methylation
	***ASXL1***	18.20%	PR-DUB component	G646fs(443/7538)	Gain of function
	***EZH2***	5.50%	H3K27 methyltransferase	R690H/C(28/7538), D664N/E/H(16/7538)	Loss of function
	***SRSF2***	13.10%	RNA splicing, splice site selection	P95H/L/R(930/7538)	Different alternative splicing
	*SF3B1*	14.90%	Pre-mRNA splicing	K700E(610/7538), K664N/R/T/Q/E/M(189/7538)	Altered 3’-splice recognition site
	*RUNX1*	11.60%	Transcription factor	R201Q/*/G(79/7538), R166*/Q/G/L(66/7538)	Loss of function
	*TP53*	10.50%	Tumor suppressor	R273H/C/G(77/7538), R248Q/W/L/P(69/7538)	Loss of function
AML	*FLT3*	29.80%	Tyrosine kinase	E611_F612 ins(120/1567), D835Y/E/H/V/N(88/1567)	Gain of function
	*NPM1*	24.50%	Nucleophosmin	W288Cfs*12(323/1567)	Loss of function
	*DNMT3A*	22.50%	DNA methyltransferase	R882H/C/P(185/1567)	Loss of function
	*NRAS*	12.30%	GTPase	G12D/S/C/A/R(68/1567), G13D/R/V/C(60/1567), Q61K/H//P/R/L(68/1567)	Gain of function
	*TET2*	12.30%	DNA demethylase	R882H/C/P(739/1567)	Loss of function
	*IDH2*	11.90%	Isocitrate dehydrogenase	R140Q/L/W(151/1567)	Gain of function
	*RUNX1*	11.60%	Transcription factor	R162K/G/S/W(23/1567), R201*/Q(17/1567)	Loss of function
	***SRSF2***	10.50%	RNA splicing factor	P95H/L/R(158/1567)	Different alternative splicing
	*TP53*	8.80%	Tumor suppressor	R248Q/W(19/1567), R273H/C/S(15/1567)	Loss of function
	***ASXL1***	8.50%	PR-DUB component	G646Wfs*12/4Wfs*12(46/1567), G645Vfs*58/Wfs*12(24/1567)	Gain of function

Mutation data were derived from cBioPortal (accessed April 2025). MDS datasets include: Yoshida et al., Nature 2011 (mds_yoshida_2011); *MSK cohort*, 2020 (mds_msk_2020); Bernard et al., NEJM Evidence 2022, IPSS-M cohort (mds_ipssm_2022). AML datasets include: Tyner et al., Cancer Cell 2022 (aml_ohsu_2022); Papaemmanuil et al., Nature 2018 (aml_ohsu_2018); *TCGA PanCancer Atlas* (laml_tcga_pan_can_atlas_2018). Mutation frequencies represent the number of patients harboring at least one mutation in the indicated gene divided by the total number of profiled patients per dataset, as reported in cBioPortal. In the AML cohort, the inability to distinguish secondary AML cases arising from antecedent MDS from de novo AML may underlie the observed discrepancies in gene mutation frequencies between the MDS and AML datasets. Hotspot mutations were manually selected as the most frequent or representative protein-level variants for each gene across the selected cohorts. These represent the number of patients with the specific mutation over the total number of profiled patients.

*MDS* myelodysplastic neoplasia, *AML* acute myeloid leukemia, *PR-DUB* polycomb repressive deubiquitinase.

This review aims to provide a consolidated update on recent research with a focus on adverse MDS/AML risk factors, specifically ASXL1, SRSF2, and EZH2. Other factors, such as DNMTs and TETs are well-reviewed elsewhere [8–11].

## Chromatin and RNA splicing dysregulation in leukemia

Chromatin dysregulation refers to pathological alterations in chromatin structure and function, often driven by mutations in epigenetic regulators, that lead to abnormal gene expression and disease. In leukemia, such as MDS/AML, chromatin dysregulation plays a critical role through mutations in key DNA and histone modifiers, for example TET2, DNMT3A, ASXL1, and EZH2, which as mentioned above are frequently mutated in MDS/AML (Table 1). DNA methylation and histone modifications are major factors in transcription regulation. The degree of DNA methylation at promoters and enhancers dictates transcription control, with elevated levels impeding the process. In MDS/AML, the crucial balance of DNA methylation and demethylation is largely disrupted due to mutations in DNMT and TET enzymes. DNMT3A functions as a DNA methyltransferase, while TET2 facilitates DNA demethylation. Meanwhile, EZH2, a component of the Polycomb repressive complex 2 (PRC2), and ASXL1, a component of the Polycomb repressive deubiquitinase (PR-DUB), are polycomb group (PcG) proteins that play critical roles in gene silencing, primarily through histone modifications (Fig. 1, right). Interactions between PRC1 and PRC2 have been well-documented; PRC1 facilitates ubiquitination at H2AK119 (H2K119ub1), and thereby recruits the PRC2 complex to chromatin. PRC2 in turn catalyzes methylation at H3K27 (H3K27me3), promoting a transition from euchromatin to heterochromatin and hence recruitment of PRC1. Contrasting with this, the PR-DUB complex containing ASXL1 opposes the action of PRC1 by removing ubiquitin from H2AK119ub [12, 13]. This removal indirectly leads to decreased H3K27me3 level by attenuating the interaction between PRC1 and PRC2. Thus, EZH2 and ASXL1 are proteins that control two histone modifications, H3K27me3 and H2AK119ub1, essential for chromatin condensation (Fig. 1).

RNA splicing factors are also vital contributors in regulation of gene expression at the post-transcriptional level. These factors accumulate in specific regions of the nucleus, forming nuclear speckles where transcription is highly activated [14, 15] (Fig. 1, left). Mutations in splicing factors not only affect proper RNA splicing but also have significant impacts on nuclear speckle formation and 3D chromatin interactions [16, 17]. Among these factors, SRSF2 and SF3B1 are prominently associated with leukemia, as detailed in Table 1; previous studies have linked mutations in these splicing factors to adverse prognoses, primarily through alteration of the RNA splicing mechanism [18–21]. However, the precise mechanisms by which such mutations influence disease progression remain to be fully elucidated.

## The PR-DUB complex: ASXL1 mutations and their impact on leukemogenesis

The PR-DUB complex, consisting of the subunit proteins ASXL1 and BAP1, is crucial in the removal of H2AK119ub. BAP1 is the catalytic subunit of PR-DUB, while ASXL1 is essential for its activity. By counteracting the activity of PRC1 through the deubiquitination of H2AK119Ub, PR-DUB facilitates the transition of chromatin from a heterochromatin state to a euchromatin state, thereby facilitating gene expression (Fig. 1, right).

**Fig. 2:**
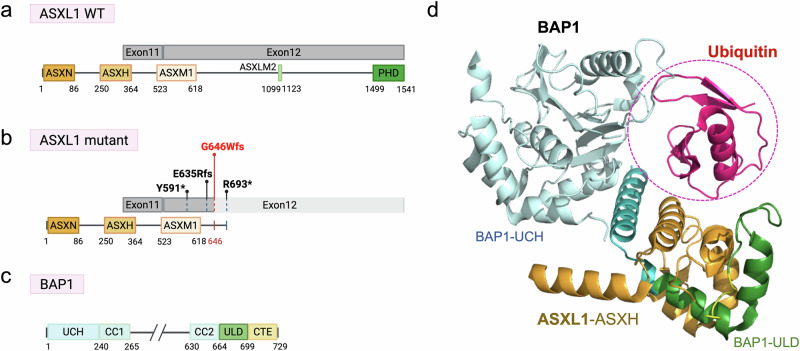
Domain architecture of PR-DUB components and frequent mutations in ASXL1. **a** Diagram of major ASXL1 domains. ASXN ASX N-terminal domain, ASXH BAP1 binding domain, ASXM1/2 ASX middle domain 1/2, PHD Plant homeodomain. **b** Locations of ASXL1 truncation mutations recurrently found in MDS/AML. The majority occur in exon 12, with G646Wfs recognized as the most frequently occurring variant (indicated in red). The relative frequency of each mutation is represented by the length of the line. Y591*: Nonsense mutation at position 591 results in a premature stop codon, replacing tyrosine. E635Rfs: Deletion of several nucleotides causes a glutamine-to-arginine substitution at position 635, leading to a frameshift and the generation of a premature stop codon. G646Wfs: Insertion of a single nucleotide causes a glycine-to-tryptophan substitution at position 646, leading to a frameshift and the generation of a premature stop codon. R693*: Nonsense mutation at position 591 results in a premature stop codon, replacing tyrosine. **c** Diagram of BAP1 domains. UCH domain: Ubiquitin C-terminal hydrolase domain, which exhibits deubiquitinating activity essential for PR-DUB complex function; CC1/2 domain: Coiled-coil 1 and coiled-coil 2 domain; ULD domain: Ubiquitin-like domain; CTE domain: C-terminal extension. **d** The ASXH domain of ASXL1 interacts with the ULD domain of BAP1, forming a ubiquitin-binding pocket.

In leukemia, heterozygous frameshift mutations of *ASXL1* are predominantly observed. These mutations often result in the production of shortened ASXL1 proteins, with premature stop codons typically occurring around amino acid positions 591, 592, 645, 646, and 693 [22, 23] (Fig. 2a, b). Among the known mutations, the G646Wfs mutation, caused by a single nucleotide insertion and frameshift, is the most prevalent. With this mutation, approximately half of the C-terminal region is lost, suggesting that the truncated ASXL1 should lose its normal function [24, 25]. However, recent studies have indicated such truncation to confer a gain-of-function effect, as the mutated ASXL1 enhances PR-DUB enzymatic activity by stabilizing the complex. Specifically, compared to the wild-type (WT), the mutant ASXL1 reduces polyubiquitination and prevents proteasomal degradation, thereby increasing the stability of the complex [26]. As a result, the mutant ASXL1 is expressed at a level surpassing that of WT ASXL1 in leukemia, indicating a dominant-negative effect of the mutant. The consequent heightened PR-DUB activity leads to upregulation of *HOX* genes (such as *HOXA5*, *HOXA7*, and *HOXA9*), which hampers the terminal differentiation of hematopoietic stem and progenitor cells (HSPCs), contributing to decreased myeloid proliferation [26].

**Fig. 3:**
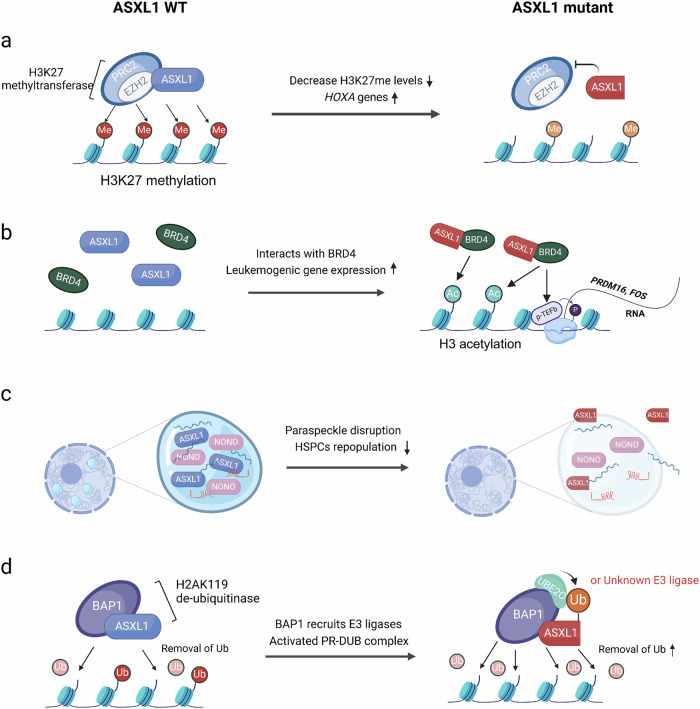
Functional impacts of ASXL1 mutations on epigenetic regulation. **a** H3K27 methylation: Wild-type ASXL1 regulates H3K27me3 through PRC2-EZH2. Mutant ASXL1 reduces H3K27me3, activating *HOXA* genes. **b** Interaction with BRD4: While WT ASXL1 does not interact with BRD4, MT ASXL1 does interact with BRD4, increasing acetylation on H3K27/122, which is associated with expression of leukemogenic genes (e.g. *PRDM16*, *FOS*). **c** Paraspeckles and HSPC repopulation: Mutation of ASXL1 disrupts paraspeckles, decreasing HSPC repopulation. **d** De-ubiquitination: Wild-type ASXL1-BAP1 removes ubiquitin from H2AK119. Mutant ASXL1 enhances this process through a gain of function.

The WT and mutant ASXL1 are known to differentially interact with other epigenetic regulators like PRC2, BRD4, and NONO [27–29]. In particular, the ASXL1 mutant exhibits reduced interaction with PRC2 that results in decreased levels of H3K27 methylation and subsequent transcriptional activation of HOX genes (e.g., *HOXA7*, *HOXA9*, *HOXA11*) [27] (Fig. 3a). Furthermore, the mutant form uniquely binds to BRD4, a member of the BET family that recognizes acetylated lysine residues [30]. This binding promotes phosphorylation of the C-terminal domain of RNA polymerase II by positive transcription elongation factor b (P-TEFb), thereby activating transcription. Consequently, histone H3 becomes acetylated at lysine residues 27 and 122 (H3K27/122ac) near the genes *PRDM16* and *FOS*, leading to their increased expression. In this way, ASXL1 mutation contributes to the upregulation of *PRDM16*, higher levels of which are associated with lower survival rates in leukemia patients [31–33] (Fig. 3b).

ASXL1 has been linked to paraspeckle formation via interacting with non-POU domain containing octamer binding (NONO) [29]. Paraspeckles are nuclear substructures composed of the lncRNA nuclear paraspeckle assembly transcript (*NEAT1*) and proteins, and are involved in post-transcriptional gene regulation, RNA processing, and cellular stress responses. Generally, wild-type ASXL1, in conjunction with NONO, upregulates *NEAT1* expression and increases the NONO-*NEAT1* interaction through the C-terminal region of ASXL1. In mutant ASXL1, this region is absent, leading to decreased interaction with NONO and hindering the formation of paraspeckles due to downregulated *NEAT1* expression (Fig. 3c). This disruption leads to RNA mis-splicing and impedes the repopulation of HSPCs, ultimately resulting in hematopoietic dysfunction.

While there are many possible explanations for how ASXL1 mutation contributes to leukemogenesis, some mechanisms remain unclear. In particular, ASXL1 mutants exhibit a unique mono-ubiquitination at the K351 site; the involvement of this modification in development of MDS/AML is unknown (Fig. 3d). In the wild-type context, this site undergoes polyubiquitination that leads to ASXL1 degradation through the proteasome pathway [34]; mono-ubiquitination at K351 in the mutant may prevent such degradation. Although the underlying mechanism is not fully determined, the increase in BAP1 activity mediated by the ASXL1 mutant might deubiquitinate ASXL1 K351ub, thereby preventing its polyubiquitination and subsequent degradation. As a result, the mutant PR-DUB (mono-ubiquitinated mutant ASXL1 and BAP1) exhibits greater structural stability and enzymatic activity compared to wild-type. In addition, Toshio Kitamura’s group identified UBE2O as the E3 ligase for mutant ASXL1 using an in vitro ubiquitination system. However, even with *UBE2O* knocked out, mono-ubiquitinated mutant ASXL1 was still observed in the condition of BAP1 co-expression, suggesting the possibility that another yet-unknown E3 ligase may act on the ASXL1 mutant. Further research into the mono-ubiquitination of ASXL1 and its role in tumorigenesis is essential for a better understanding of leukemia development and could lead to new targeted therapies.

The study of ASXL1 is limited by a lack of detailed structural information. To date, only the structure of its ASXH domain, which interacts with BAP1, has been resolved. The ASXH domain together with the UCH and ULD domains of BAP1 form a pocket for ubiquitin recognition (Fig. 2d) [35, 36]. Therefore, of ASXL1 domains, at least ASXH is required for PR-DUB activity [37, 38]. However, it remains unclear whether the C-terminal region of ASXL1, including the ASXM2 and PHD domains, contributes to ubiquitin binding and removal. Addressing this uncertainty necessitates resolving the structure of full-length ASXL1 in complex with BAP1, and would require the purification of both full-length ASXL1 and its mutants. To date, no studies have successfully purified the full-length protein. Comparative structural and biochemical analyses of full-length ASXL1 and truncated cancer-associated ASXL1 mutations could shed light on their functional consequences and the molecular mechanisms underlying ASXL1 mutations in MDS and AML. Ultimately, further research is necessary to fully elucidate the role of ASXL1 in the enzymatic activity of PR-DUB, as many aspects of its structure and function remain unclear [35–39].

## SRSF2 and SF3B1 mutations: altered RNA splicing and its impact on leukemia

Recent studies have elucidated the critical roles of spliceosome components in the pathogenesis of MDS and AML. Among these components, serine/arginine-rich splicing factor 2 (SRSF2) has recently been identified as a high-risk factor in leukemia; this protein is important for RNA binding and splice site selection [20, 40]. *SRSF2* mutations are frequently observed in MDS and AML, occurring in approximately 13.1% and 10.5% of cases, respectively, and are associated with clinical prognosis [41] (Table 1).

**Fig. 4:**
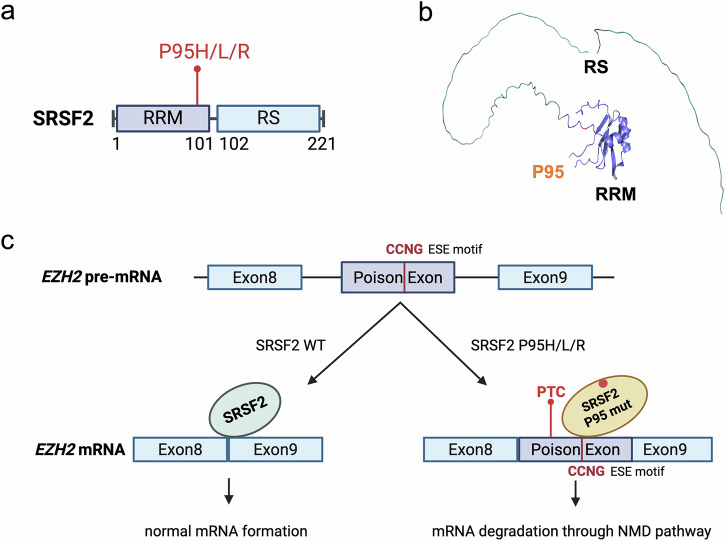
Domain architecture of SRSF2 and predicted protein structure. **a** Diagram of SRSF and the hotspot mutations at P95. P95H/L/R A proline-to-histidine, leucine, or arginine substitution at position 95. RRM RNA recognition motif, RS Arginine/serine-rich domain. **b** AlphaFold’s predicted structure for SRSF2, which indicates that P95 mutations, positioned right before the RS domain, could influence RNA-binding affinity. **c** Alternative splicing of *EZH2* mRNA in association with wild-type SRSF2 and the Pro95 mutant. The Pro95 mutant induces poison exon insertion, resulting in a premature termination codon and subsequent degradation of the mRNA through nonsense-mediated decay. The mutant SRSF2 preferentially binds to an exonic splicing enhancer (ESE) motif (CCNG) within the poison exon, thereby promoting its inclusion.

SRSF2 is a member of the serine/arginine-rich family of proteins, involved in both constitutive and alternative splicing [42]. It specifically recognizes exonic splicing enhancer motifs and promotes the assembly of spliceosomal complexes. The Pro95 residue in SRSF2 is frequently mutated, resulting in its replacement by histidine, leucine, or arginine (Fig. 4a). Structural studies have revealed that Pro95 is located within the RRM domain, positioned at the junction connecting to the RS domain. This positioning was also supported by the predicted structure from AlphaFold [43], in which the RS domain remains unresolved (Fig. 4b). Mutations at this site alter the RNA recognition motif of the RRM domain and potentially induce structural changes in the RS domain, significantly impacting the function of SRSF2. The RRM domain, known for its crucial role in recognizing target RNA, has been well-characterized in previous studies [44]. Notably, mutations at Pro95 shift the RNA-binding affinity of the protein from GGNG to CCNG, leading to altered splicing patterns of several genes. These changes have been implicated in proliferation of dysregulated hematopoietic stem cells [45, 46].

One well-studied example is the alternative splicing of *EZH2*, which encodes a catalytic subunit of PRC2. The mutation at Pro95 of SRSF2 causes inclusion of a typically-excluded poison exon in the *EZH2* mRNA, which contains a premature termination codon (Fig. 4c). This alteration triggers the nonsense-mediated decay (NMD) pathway, which degrades faulty mRNA to maintain accurate gene expression and prevent the production of defective proteins [47]. Consequently, EZH2 expression is substantially reduced, impacting hematopoiesis. In addition to *EZH2*, *JAK2, PINK1,* and *INTS3* have also been identified as targets of mis-splicing triggered by the SRSF2 Pro95 mutation [48–50], suggesting that the SRSF2 mutant may impact the mRNA splicing of yet more genes. Further investigations, such as RNA sequencing under conditions of NMD pathway inhibition or with altered exon insertion/deletion, could offer deeper insights into this mechanism.

Similar to SRSF2, SF3B1 is a recurrently mutated splicing factor in MDS and AML. Its mutations have also been associated with oncogenic processes [51], in part through mis-splicing events that disrupt chromatin regulatory mechanisms. The K700E hotspot mutation in SF3B1 promotes the inclusion of a poison exon (exon 14a) in *BRD9* transcripts, resulting in reduced BRD9 protein levels via nonsense-mediated mRNA decay (NMD). BRD9 functions as a scaffold protein within the non-canonical BAF (ncBAF) chromatin remodeling complex, and its depletion disrupts ncBAF assembly and impairs its localization to CTCF binding sites, thereby potentially altering chromatin accessibility and gene expression [52].

## The dual role of EZH2 mutations: mechanisms and implications in leukemia

Enhancer of zeste homolog 2 (EZH2) is a crucial component of PRC2, which primarily functions to mono-, di-, and tri-methylate histone H3 at lysine 27 (H3K27me3), leading to gene silencing [53]. This epigenetic modification plays a pivotal role in regulating gene expression during development and cellular differentiation [54]. Dysregulation of EZH2 activity has been implicated in various cancers, making it a significant focus of oncological research [55, 56].

Mutations in EZH2, particularly gain-of-function (GOF) mutations, have been identified in a variety of malignancies, including lymphomas, breast cancer, and prostate cancer [57–60], and frequently occur in the EZH2 SET domain [52–55]. The SET domain is the catalytic core of EZH2, responsible for its histone methyltransferase activity; specifically, this domain transfers methyl groups from the cofactor S-adenosyl-L-methionine to the lysine 27 residue of histone H3. Common GOF mutations include EZH2 Y646, A682, and A692, with the EZH2 Y646 mutation in particular being frequently reported in lymphomas such as diffuse large B-cell lymphoma and follicular lymphoma (Table 2, left). These mutations enhance EZH2 methyltransferase activity by efficiently converting H3K27me2 to H3K27me3, resulting in aberrant silencing of tumor suppressor genes and promoting oncogenesis [61, 62]. Therefore, targeting EZH2 is considered a prominent approach to treating lymphoma, and tazemetostat, which specifically targets the EZH2 SET domain, has been approved for patients with follicular lymphoma and epithelioid sarcoma [63, 64].

**Table 2:** Comparison of EZH2 mutations: Y646x, D664x, and R690x in relation to cancer and PRC2 activity.

	*EZH2* Y646x mutation	*EZH2* D664x mutation	*EZH2* R690x mutation
PRC2 activity	“Gain-of-function”	“Loss-of-function”	
Occurrence	Diffuse large B-cell lymphoma (25%)	*EZH2* mutations: 5.5% of all MDS cases, 2.9% of all AML cases	
	Follicular lymphoma (12%)		
	Melanoma (1%)		
Mutation site	EZH2 SET domain, inside the catalytic pocket	EZH2 SET domain, SET-I a-helix motif	EZH2 SET domain, close to the SAL domain
Mutation	Y646F/N/H/S/C	D664G/V/E/A/H	R690H/C/G/S
Mechanism	Increased conversion of H3K27me2 to H3K27me3	Reduce PRC2 activity	Reduce global levels of H3K27me2 and H3K27me3
Etc			Potential significance for the docking of EZH2 inhibitors

Despite EZH2 being an established target in lymphoma therapy, further research remains needed to clarify the role of frequently observed EZH2 mutations in MDS and AML. Unlike gain-of-function mutations, these alterations are typically associated with a loss-of-function mechanism [65, 66]. EZH2 mutations occur in 5.5% of MDS patients (Table 1, 2), predominantly in a heterozygous state, with homozygous mutations being rare. Studies indicate that homozygous mutations may impact survival, while heterozygous mutations are commonly associated with both lymphomas and leukemias [67]. Our investigation in the overall leukemia patient dataset reveals the most frequently observed EZH2 mutations to be R690 and D664 (Table 2), both of which are loss-of-function mutations [68–70]; both are located in the SET domain (catalytic domain) and are directly associated with inactivation of PRC2.

The R690 mutation in EZH2 is the second most common mutation observed in cancer, and the affected residue is critical to the docking of EZH2 inhibitors targeting the catalytic SET domain of EZH2 [71]. Previous research has demonstrated this mutation to result in lower activity compared to wild-type PRC2 [70]. Similarly, the D664 mutation was predicted to result in reduced activity based on structural studies [69]. Our histone methyltransferase assay results also indicate the PRC2-EZH2 complex containing the EZH2 D664V mutant to exhibit a significant reduction in transferase activity (data not shown). Unlike the Y646 mutation, which is the most common and well-documented as a gain-of-function mutation in lymphomas, the mechanisms underlying the R690 and D664 mutations are not well understood. Therefore, further research is required to elucidate in detail how the R690 and D664 mutations affect EZH2 function and to determine whether the R690 mutation confers resistance to EZH2 inhibitors.

## Clinical implications of ASXL1, EZH2, and SRSF2 mutations in high-risk MDS/AML

Mutations in *ASXL1*, *SRSF2*, and *EZH2* are classified as MDS-related gene mutations. The presence of any of these mutations in a patient with AML is considered evidence of AML-MRC (myelodysplasia-related change), regardless of any prior history of MDS. Additionally, these mutations are now recognized as adverse risk factors and incorporated into the standard risk stratification system recommended by the ELN 2022 guidelines [5].

*ASXL1* mutations are frequently detected in clonal hematopoiesis [72] and are known to drive myeloid transformation [22]. Consequently, such mutations are commonly observed in myeloid malignancies, including chronic myeloid leukemia [73], chronic myelomonocytic leukemia [74], myeloproliferative neoplasm, and high-risk MDS/AML. *ASXL1* mutation is believed to be an early mutational event that confers a competitive growth advantage to HSCs in various myeloid malignancies [72], even in the early stages of idiopathic cytopenia of undermined significance [75], a condition characterized by unexplained low blood cell counts without a definitive diagnosis of hematologic malignancy.

**Fig. 5:**
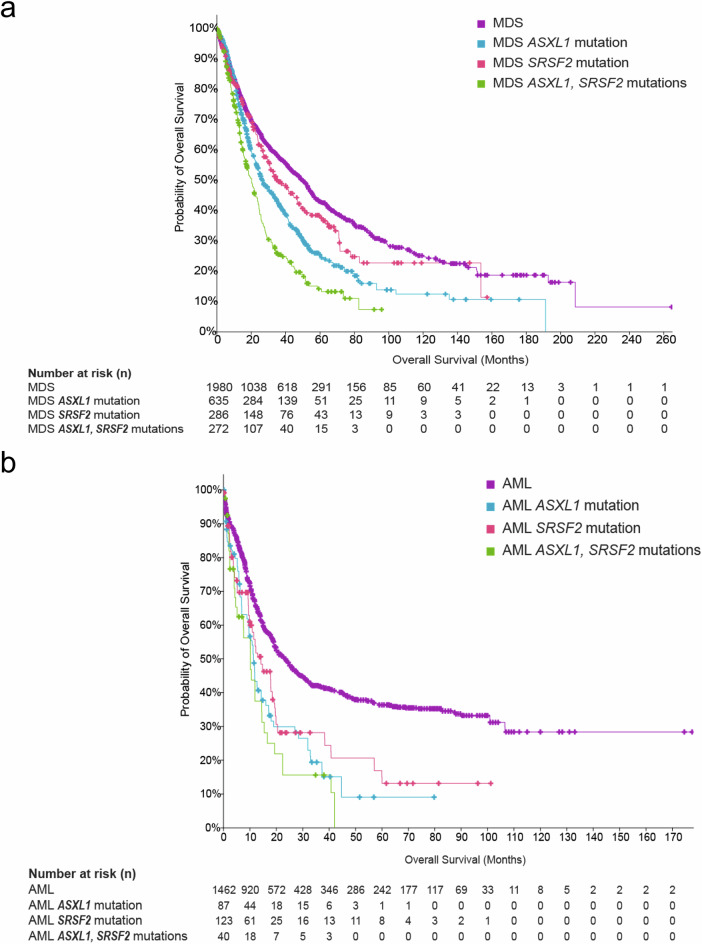
Patient overall survival in relation to *ASXL1* and *SRSF2* mutation in MDS and AML. **a** Using data sets from UTokyo (*Nature* 2011), MSK (2020), MDS IWG (IPSSM, *NEJM Evidence* 2022), and CIRM (*NEJM* 2013), MDS patients were categorized based on the presence of *ASXL1* and *SRSF2* mutations. Patients with *ASXL1* and *SRSF2* co-mutations exhibit a distinct survival curve compared to other groups. **b** Using data sets from OHSU (*Cancer Cell* 2022, *Nature* 2018), TCGA (Firehose Legacy), and TARGET (2018), AML patients were categorized based on the presence of *ASXL1* and *SRSF2* mutations. Patients harboring *ASXL1* and *SRSF2* co-mutations exhibit significantly lower survival compared to those without these genetic alterations. The MDS or AML group comprises patients without mutation in either gene.

The presence of *ASXL1* mutations consistently serves as an adverse prognostic factor in myeloid malignancies, including MDS/AML. Indeed, multiple studies have identified *ASXL1* mutation as an independent predictor of poor survival in AML [76] (Fig. 5). A role for such mutation as a poor prognostic marker has also been suggested in MDS [77].

Of splicing factor genes, mutation in *SF3B1* is associated with a distinct subtype of MDS with ringed sideroblasts (MDS-RS). In contrast, *SRSF2* mutations are not restricted to any specific myeloid neoplasm but are associated with altered myeloid differentiation, disrupting other genes and thereby causing distinctive clinical manifestations of myelofibrosis, monocytosis, and blast phase [78]. *SRSF2* mutations are also linked to poor overall survival in both AML and MDS (Fig. 5).

*SRSF2* mutations are abundant in ASXL1-mutated MDS/AML patients, with a frequency of 34.4% in ASXL1-mutated AML vs. 7.8% in non-mutated AML, and 28.5% in ASXL1-mutated MDS vs. 9.7% in non-mutated MDS (cBioPortal database). This suggests a possible correlation between the two mutations. Several studies have highlighted co-mutation of *ASXL1* and *SRSF2* in subsets of myeloid neoplasms [79–81], for which the clinical implications remain unclear. Nonetheless, the clinical challenges posed by ASXL1 and SRSF2 mutations are further supported by mechanistic evidence. A study by Wang et al. further illustrated the pathogenic cooperation between ASXL1 and SRSF2 mutations. In a murine model, sequential acquisition of ASXL1 and SRSF2 mutations recapitulated features of clonal hematopoiesis and induced progression to MDS/AML, highlighting the biological basis for the adverse clinical outcomes associated with these mutations [82].

*TET2* and *DNMT3A* are the most commonly mutated genes in MDS and AML. Currently, leukemia patients are often treated with DNA hypomethylating agents due to the frequent epigenetic alterations observed in these diseases. However, effective treatment methods targeting ASXL1 and SRSF2 still need to be developed. Current standard treatment for higher-risk MDS/AML has limited efficacy in patients with mutation of *ASXL1* and/or SRSF2, which has led to these mutations being classified as adverse risk factors in the ELN 2022 recommendations [5]. Recent studies have explored alternative therapeutic strategies to address this gap. One such study found that venetoclax, a BCL-2 inhibitor approved for newly diagnosed AML in combination with hypomethylating agents as a less-intensive induction regimen, may mitigate the poor prognosis associated with SRSF2 mutations in AML [83]. Another study demonstrated that adding a CDK9 inhibitor to conventional hypomethylating therapy may be effective in ASXL1-mutated high-risk MDS/AML [84]. In line with these efforts, recent studies have investigated the potential of PRMT5 inhibition as a therapeutic approach for patients harboring splicing factor mutations such as SRSF2, SF3B1, and U2AF1. PRMT5 is known to modulate mRNA splicing via methylating several splicing factors [85, 86]. Although clinical responses were limited, a phase I trial of GSK3326595 reported clinical benefit in a small subset of SRSF2-mutated cases [87]. A subsequent study using PRT543, a PRMT5 inhibitor, expanded the cohort to include additional splicing factor-mutated patients and observed marrow complete remission or disease stabilization in some individuals [88]. Although PRMT5 plays a critical role in mRNA splicing, its inhibition has been shown to suppress the growth of cancers with splicing factor mutations. This effect may arise either from impaired splicing factor methylation or from disruption of other PRMT5-dependent functions. Another possibility is that the combined disruption of mRNA splicing caused by both SRSF2 mutations and PRMT5 inhibition may lead to synthetic lethality. These hypotheses warrant experimental validation in the near future. Together with other studies, these findings support emerging therapeutic strategies aimed at mitigating the adverse effects of ASXL1 and SRSF2 mutations in MDS/AML.

Development of novel agents directly targeting ASXL1 and/or SRSF2 mutations represents another potential approach. A previous study demonstrated the efficacy of a spliceosome inhibitor in SRSF2-mutated leukemia in a murine model [89]. A deeper understanding of the pathophysiology of ASXL1 and SRSF2 mutations may usher in an era of targeted therapies for patients with ASXL1/SRSF2-mutated MDS/AML.

*EZH2* mutations are also listed as adverse genetic factors in the 2022 ELN recommendations [5], and are enriched in the ASXL1-mutated population compared to ASXL1 wild-type patients with MDS [90]. An EZH2 inhibitor has been tested in patients with relapsed or refractory B-cell lymphoma and advanced solid tumors, showing a favorable safety profile [91], though it has not yet been tested in MDS/AML as the majority of cases harbor loss-of-function *PRC2* mutations. As our understanding of these epigenetic factors grows, it may lead to more targeted therapies and improved treatment strategies for patients with MDS/AML in the future.

## Conclusions and perspective

**Fig. 6:**
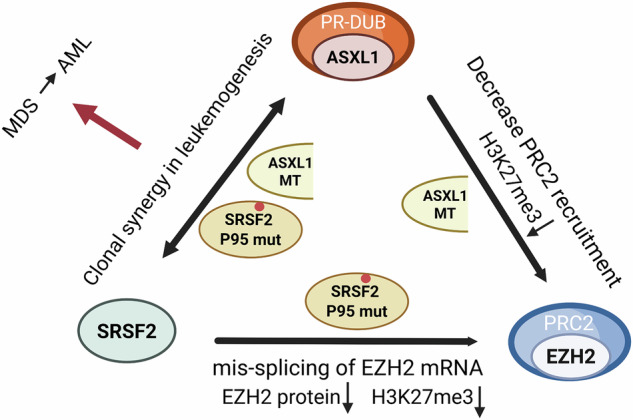
Convergent epigenetic dysregulation by ASXL1, SRSF2, and EZH2 mutations. Mutations in ASXL1 and SRSF2 are frequently co-occurring in MDS and AML and cooperatively contribute to disease progression. ASXL1 mutations enhance PR-DUB complex activity, leading to excessive H2AK119 deubiquitination and subsequent impairment of EZH2-PRC2 recruitment and reduced H3K27me3 levels. Concurrently, the SRSF2 Pro95 mutant induces mis-splicing of EZH2 mRNA, resulting in decreased EZH2 protein expression and further attenuation of H3K27me3. These alterations collectively impair Polycomb-mediated gene repression and disrupt hematopoietic regulation. The triangle highlights the clonal synergy between ASXL1 and SRSF2 mutations, converging on EZH2 dysfunction and epigenetic deregulation in leukemogenesis.

The progression from MDS to AML occurs as mutations accumulate in epigenetic regulators and splicing factors, leading to disruptions in chromatin homeostasis and abnormal gene expression. Key genes involved in this process include *ASXL1*, *SRSF2*, and *EZH2*, alongside *DNMT3A* and *TET2* (Fig. 6). These chromatin regulators possess interconnected functions, with each mutation contributing to leukemia progression and severity. Accordingly, co-mutations, such as of *ASXL1* with *SRSF2* or *EZH2*, synergistically exacerbate disease severity [78, 81, 90]. ASXL1 and EZH2 jointly regulate repressive histone modifications such as H3K27 methylation and H2A K119 ubiquitination, while the SRSF2 Pro95 mutant causes mis-splicing of *EZH2* mRNA [12, 20, 65, 66]. Although arising via distinct mechanisms—SRSF2-induced mis-splicing and EZH2 loss-of-function mutations—both events ultimately reduce EZH2 protein levels and H3K27me3 [46, 66], leading to attenuation of PRC2-mediated repression. This allows derepression of target genes involved in tumor suppression and lineage control, thereby disrupting hematopoietic regulation and promoting leukemogenesis. Such convergence may partly explain the more aggressive clinical behavior observed in SRSF2 and EZH2 co-mutant cases. Understanding the intricate crosstalk between these regulators and their molecular consequences remains pivotal to unraveling the pathogenesis of MDS and AML. Greater knowledge in this area will pave the way for precision-targeted therapies, offering a path forward to combat high-risk mutations and overcome therapeutic resistance. Bridging these mechanistic insights with innovative treatments could ultimately transform patient outcomes and redefine the clinical landscape for MDS and AML.
